# Supplementary material for: Retinal Neuron Is More Sensitive to Blue Light-Induced Damage than Glia Cell Due to DNA Double-Strand Breaks
Source: Cells. 2019 Jan 18;8(1):68. doi: 10.3390/cells8010068 (PMC6356720; doi:10.3390/cells8010068)
Supplement: Supplementary file 1 [file cells-08-00068-s001.zip › cells-409296-SI.pdf]

Supplemental data

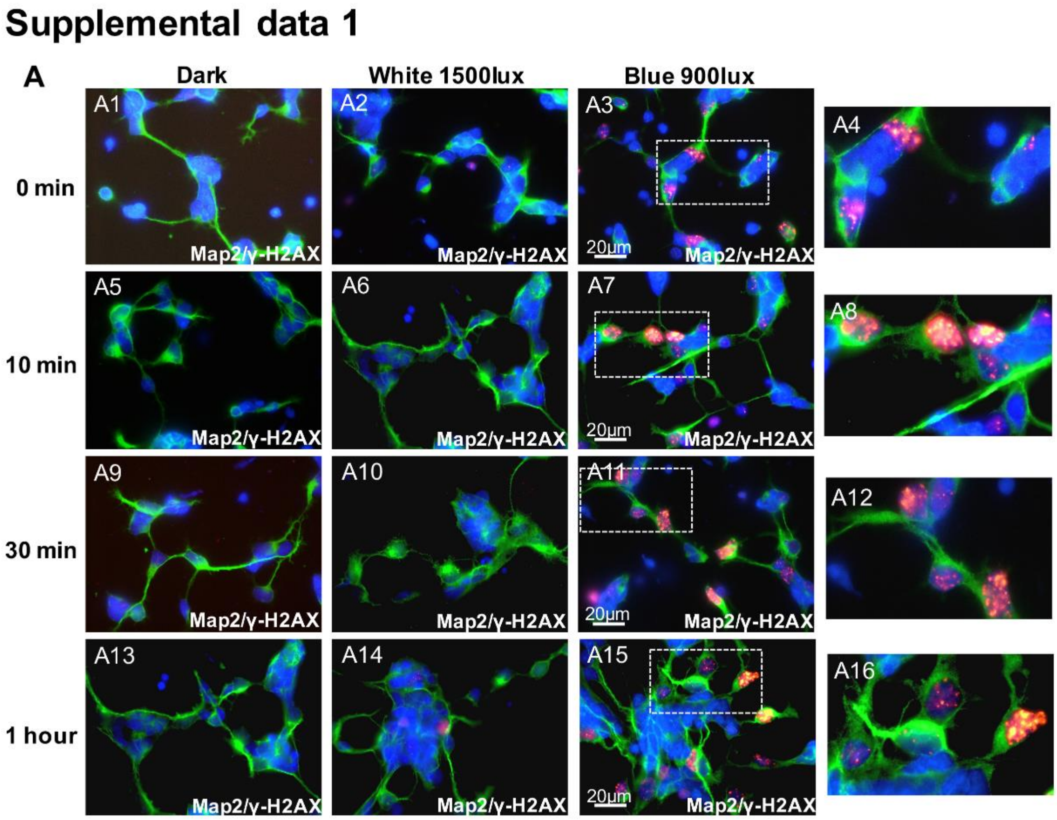

Figure S1: γ-H2AX foci label DNA double-strand breaks in retina neurons. The double staining assay were performed in retina cells 0 minute, 10 minutes, 30 minutes, 1 hour after light treatment (dark, white, blue). **A1-A3,** Double staining for Map2 and γ-H2AX in retinal neurons 0 minute after light treatment. **A4,** The magnification of the γ-H2AX foci in A3 (white dotted box). **A5-A7,** Double staining for Map2 and γ-H2AX in retinal neurons 10 minute after light treatment. **A8,** The magnification of the γ-H2AX foci in A7 (white dotted box). **A9-A11,** Double staining for Map2 and γ-H2AX in retinal neurons 30 minute after light treatment. **A12,** The magnification of the γ-H2AX foci in A11 (white dotted box). **A13-A15,** Double staining for Map2 and γ-H2AX in retinal neurons 10 minute after light treatment. **A16,** The magnification of the γ-H2AX foci in A15 (white dotted box).

Supplemental data 2

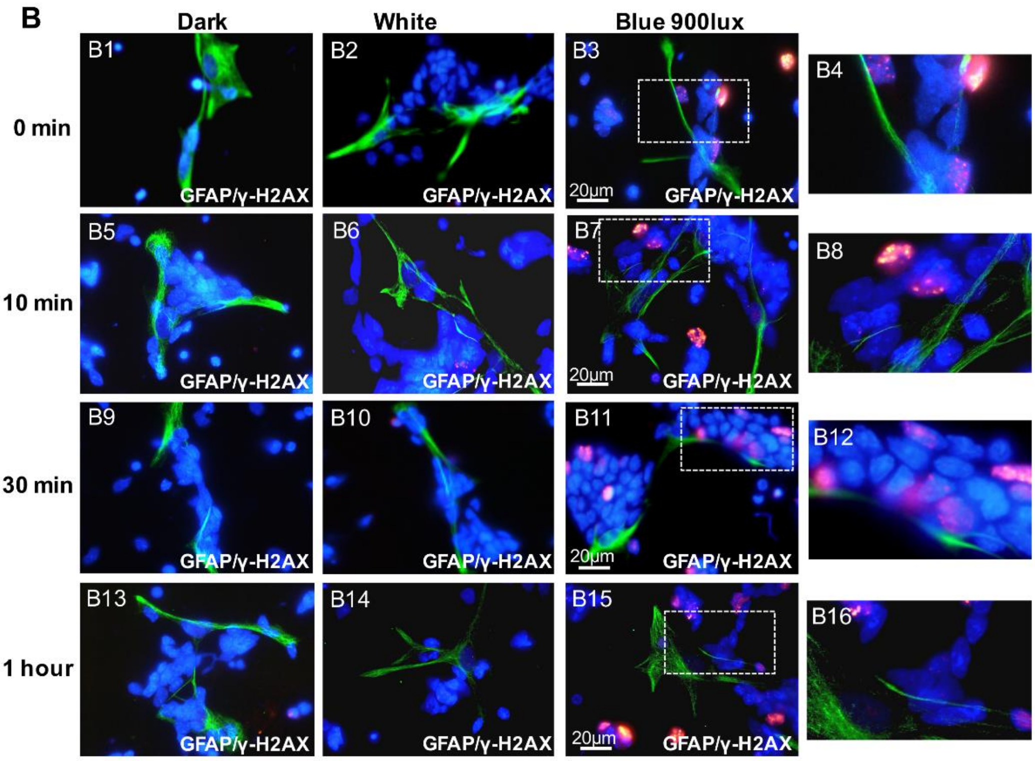

Figure S2. γ-H2AX foci label DNA double-strand breaks in glia cells. The double staining assay were performed in glia cells 0 minute, 10 minutes, 30 minutes, 1 hour after light treatment (dark, white, blue). **A1-A3,** Double staining for GFAP and γ-H2AX in glia cells 0 minute after light treatment. **A4,** The magnification of the γ-H2AX foci in A3 (white dotted box). **A5-A7,** Double staining for GFAP and γ-H2AX in glia cells 10 minute after light treatment. **A8,** The magnification of the γ-H2AX foci in A7 (white dotted box). **A9-A11,** Double staining for GFAP and γ-H2AX in glia cells 30 minute after light treatment. **A12,** The magnification of the γ-H2AX foci in A11 (white dotted box). **A13-A15,** Double staining for GFAP and γ-H2AX in glia cells 10 minute after light treatment. **A16,** The magnification of the γ-H2AX foci in A15 (white dotted box).
